# Supplementary material for: Mapping of Outdoor Food and Beverage Advertising around Spanish Schools
Source: Nutrients. 2022 Jul 31;14(15):3167. doi: 10.3390/nu14153167 (PMC9370640; doi:10.3390/nu14153167)
Supplement: Supplementary file 1 [file nutrients-14-03167-s001.zip › nutrients-1783149-supplementary.pdf]

Supplementary File S1

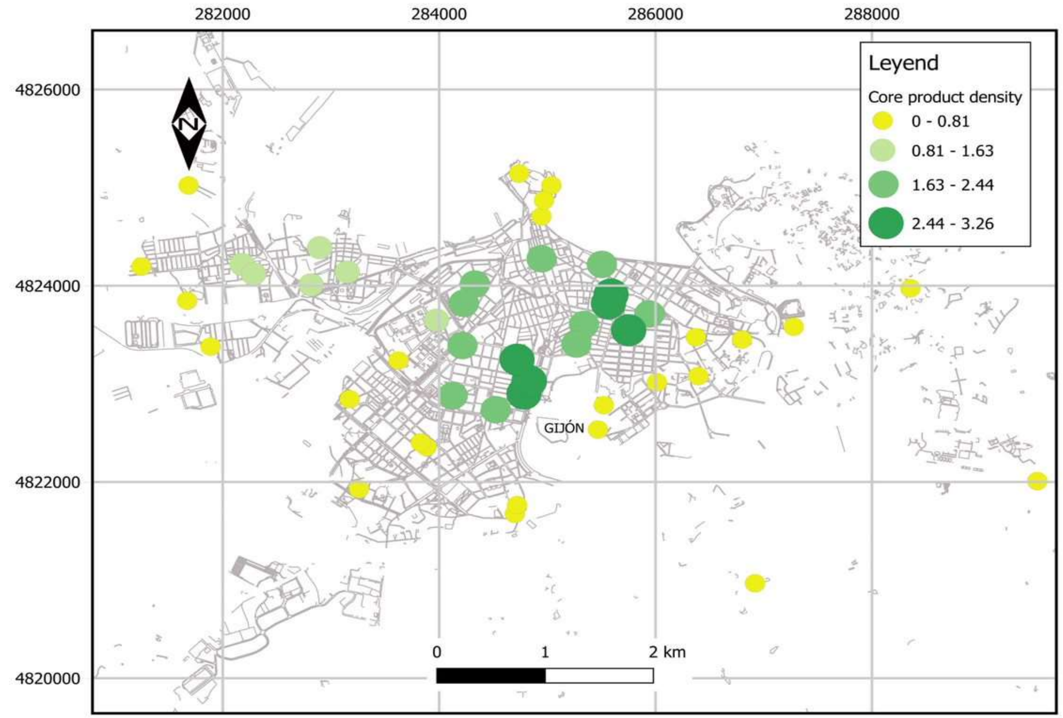

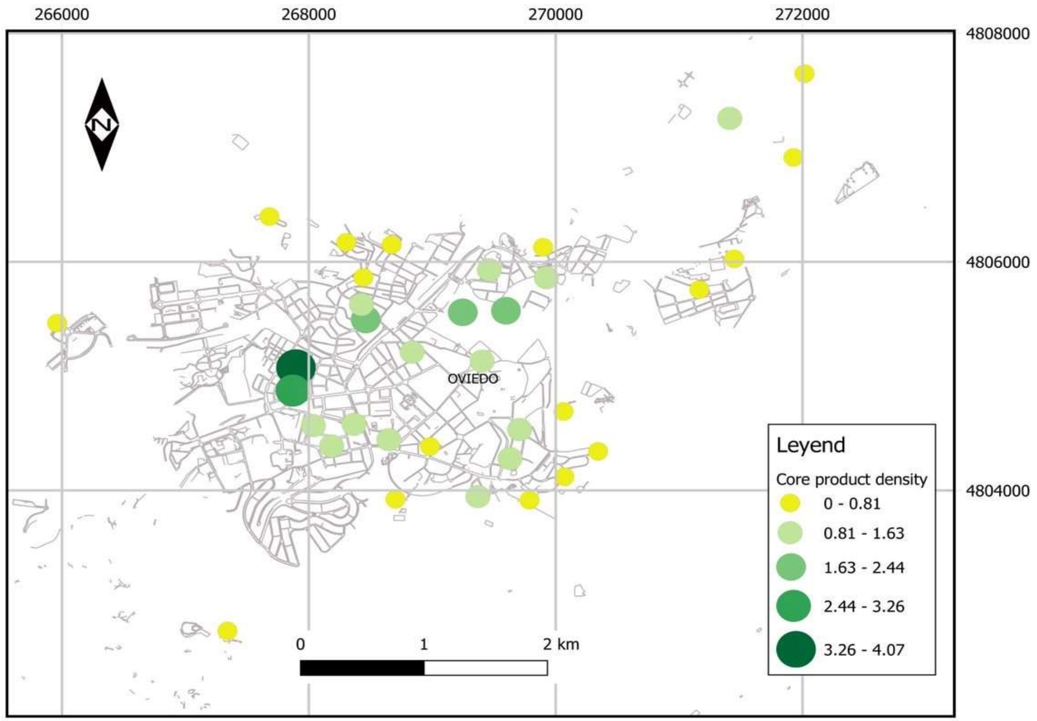

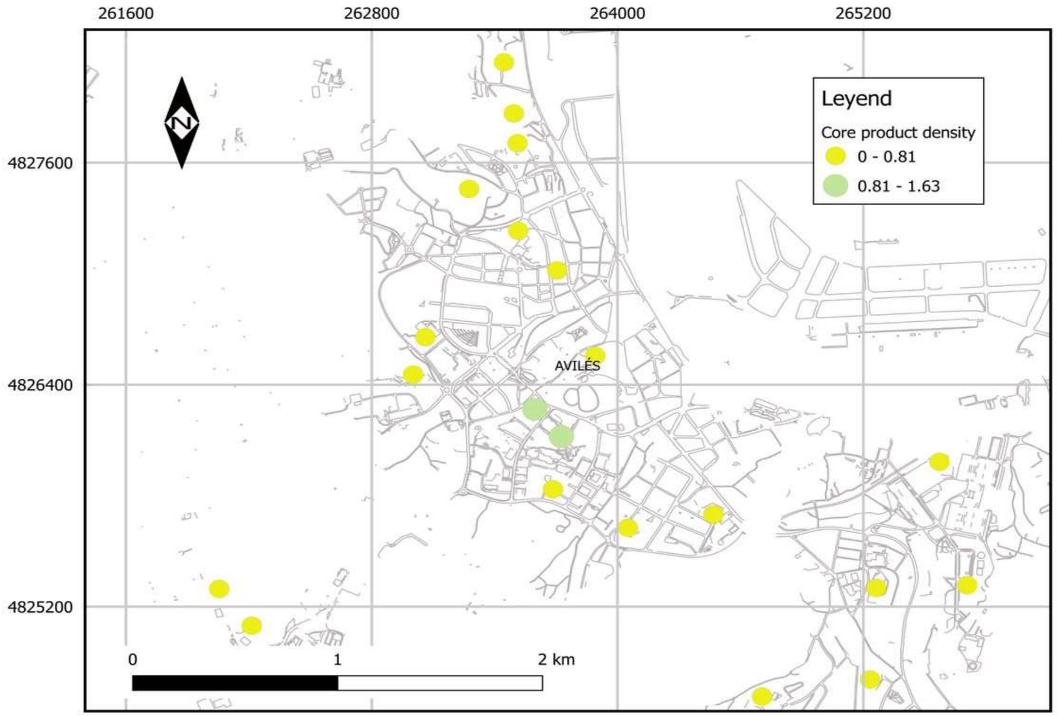

Figure S1. Density (x10-4points/m2) of core products advertised in Gijón, Oviedo, and Avilés respectively. Coordinate reference system ETRS89, UTM 30N projection.

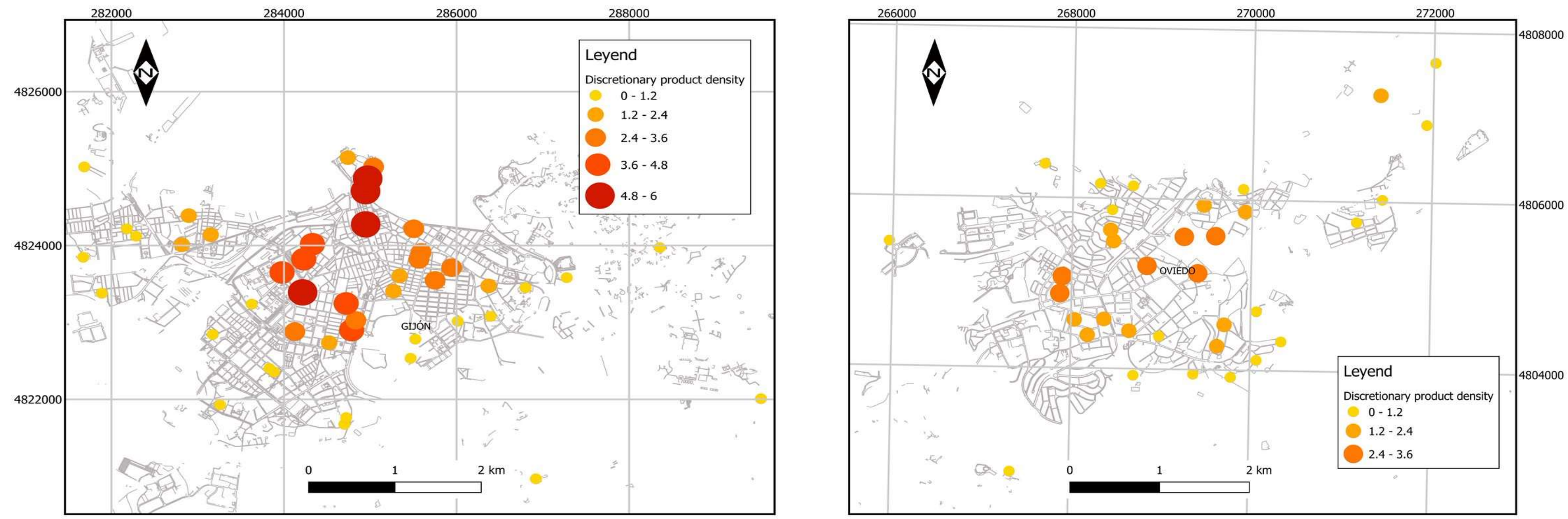

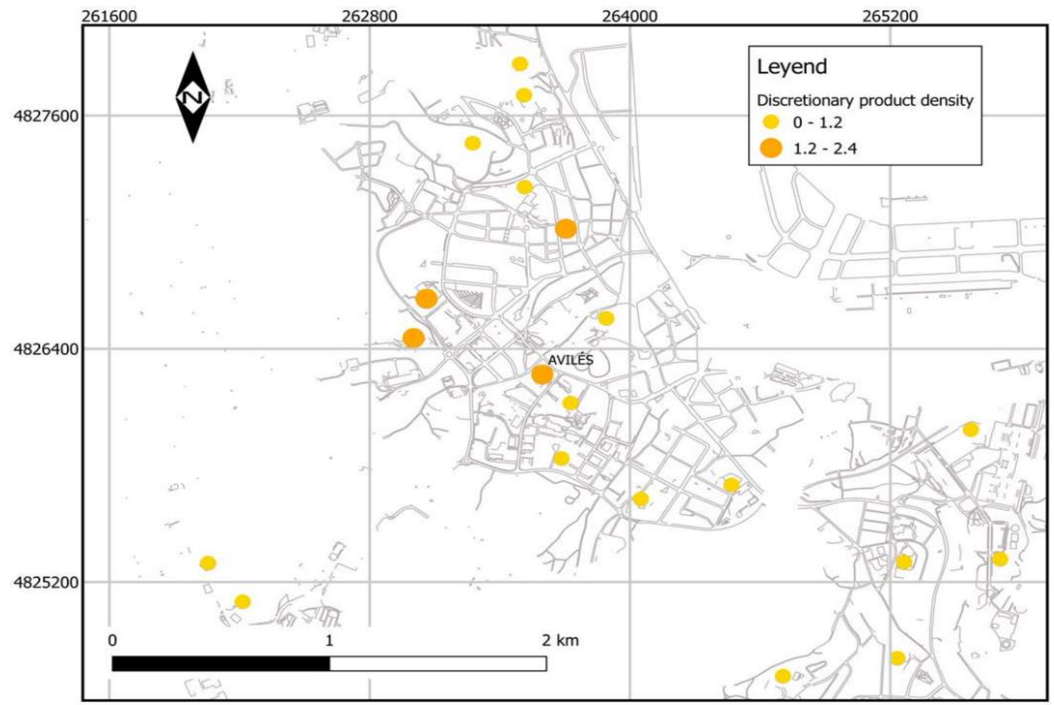

Figure S2. Density (x10-4points/m2) of discretionary products advertised in Gijón, Oviedo and Avilés respectively. Coordinate reference system ETRS89, UTM 30N projection.

Supplementary file S2: products classification based on previous recommendations [19-21]

**Core/healthy**	Grains	Rice, pasta, bread, breakfast cereals (< 30 g/100 g sugar or < 35 g/100 g sugar if includes fruit), savoury biscuits (energy < 1800 KJ/100 g)
	Fruits	Fresh, 100% fruit juice
	Vegetables	Fresh, frozen, pre-packaged vegetable mixes, tomato puree and pastes, olives, vegetables cream
	Dairy and alternatives	Milk, all yoghurt, cheese, cream cheese
	Meat and alternatives	Fresh/frozen/roasted, beef, pork, chicken, fish, canned fish, smoked fish, seafood products, eggs
	Oil	Olive oil, sunflower oil
	Water	Unflavored mineral water
**Discretionary/ unhealthy**	Processed meat	Sausages, hamburgers, bacon, processed delicatessen meats
	Chips and savory snacks	Chips and savory snacks, not sweet
	Fruit beverages	Fruit juice (not 100%, sugar added)
	Other beverages	Full sugar soft drinks, sugar added drinks, flavored milks, sport drinks, electrolyte drinks
	Desserts and Ice cream	Cake, sweet biscuits, scones, canned fruit in syrup, frozen yoghurt, sweet breads, pastries, doughnuts, ice cream, Breakfast cereals (> 30 g/100 g sugar or > 35 g/100 g sugar if includes fruit), savoury biscuits (energy > 1800 KJ/100 g), snack bars, jam, nougat
	Chocolate and candy	Chewing gum, gummies, chocolate products, chocolate toppings, chocolate bars, sweetened cocoa
	Unhealthy ready meals, salt and fat products	Fast food, daily menu, pizza, burgers, nuggets, snack, sandwich, ready to eat and frozen meals and other salt or fat products (margarine, butter)
	Soft and energy beverages	Diet, sugar-free soft drinks, energy drinks
	Alcohol	Alcohol beverages (Beer, wine, spirits, cider…)
**Others/ Miscellaneous**	Recipe additions	Herbs, spice, sauces
	Tea and coffee	Tea, infusions and coffee

Supplementary material S3. Density of core and discretionary products by city and school radius

Core Products

Gijón

School code	Density (nx10-4(points/m2))
33005350	0.43
33006342	0.00
33028601	0.00
33005362	1.18
33020545	1.20
33020557	0.56
33022554	1.04
33022578	1.06
33020570	1.03
33022104	0.55
33006445	0.14
33021914	0.29
33023601	1.99
33022566	0.29
33020314	0.15
33005404	1.08
33006007	2.42
33021926	1.74
33005957	1.97
33005969	2.38
33006081	0.20
33005428	2.98
33021872	0.04
33006494	0.04
33021653	2.72
33019701	2.66
33028982	1.66
33022335	0.38

33006093	0.33
33005945	0.00
33006287	0.27
33005891	2.16
33005982	2.42
33005933	1.77
33027874	0.05
33005398	2.83
33005908	3.08
33005881	1.85
33024289	2.57
33005911	0.36
33005337	0.17
33005878	0.51
33021938	0.20
33006044	0.00
33021665	0.04
33006184	0.01
33005210	0.00

Oviedo

**School code**	**Density (nx10-4(points/m2))**
33013085	0.04
33012721	0.00
33012731	0.00
33012664	4.07
33021781	3.11
33022581	1.69
33012378	1.11
33013103	0.14
33013152	1.41
33012639	2.27
33012688	0.37
33012457	1.53
33012755	0.25

33012408	0.98
33012767	0.27
33012652	0.94
33012640	0.61
33012421	1.86
33012411	1.16
33022281	0.92
33012627	1.03
33022347	1.86
33023613	1.12
33012743	0.89
33012573	0.99
33013097	0.45
33012691	0.95
33012676	0.47
33012433	0.42
33020909	0.24
33021471	0.52
33012044	0.85
33012536	0.18
33028672	0.05
33028064	0.13

Avilés

**School code**	**Density (nx10-4(points/m2))**
33001216	0.00
33001472	0.00
33001319	0.67
33022098	0.62
33001022	0.00
33001344	0.43
33001046	0.00
33001137	0.89
33001228	0.00
33020132	0.51

33001034	0.65
33001435	1.08
33001186	0.22
33001095	0.69
33022086	0.46
33001149	0.33
33001174	0.20
33001150	0.27
33022256	0.08
33001460	0.20
33001231	0.20

Discretionary products

Gijón

School code	Density (nx10-4(points/m2))
33005350	0.45
33006342	0.25
33028601	0.06
33005362	0.78
33020545	1.03
33020557	0.50
33022554	1.59
33022578	1.57
33020570	1.68
33022104	0.60
33006445	0.24
33021914	0.79
33023601	5.90
33022566	0.79
33020314	1.07
33005404	3.82
33006007	3.11
33021926	4.39
33005957	4.23
33005969	2.60
33006081	2.30
33005428	4.21
33021872	0.15

33006494	0.15
33021653	3.91
33019701	3.60
33028982	6.47
33022335	5.53
33006093	5.09
33005945	0.00
33006287	2.95
33005891	1.91
33005982	1.87
33005933	3.21
33027874	0.13
33005398	3.30
33005908	3.25
33005881	3.31
33024289	2.98
33005911	1.12
33005337	0.51
33005878	1.69
33021938	0.71
33006044	0.00
33021665	0.18
33006184	0.03
33005210	0.00

Oviedo

**School code**	**Density (nx10-4(points/m2))**
33021471	0.97
33012044	2.51
33012536	0.39
33028672	0.24
33028064	0.71
33013085	0.09
33012721	0.00
33012731	0.01
33012664	3.11
33021781	2.98
33022581	1.99
33012378	1.71
33013103	0.33
33013152	1.90

33012639	2.19
33012688	0.97
33012457	1.80
33012755	0.62
33012408	1.54
33012767	0.39
33012652	2.97
33012640	0.80
33012421	3.40
33012411	2.95
33022281	1.04
33012627	1.66
33022347	3.44
33023613	2.02
33012743	1.77
33012573	1.13
33013097	0.92
33012691	1.77
33012676	0.83
33012433	1.09
33020909	0.36

Avilés

**School code**	**Density (nx10-4(points/m2))**
33001216	0.00
33020132	1.52
33001034	0.97
33001435	1.09
33001186	0.81
33001095	1.15
33022086	1.06
33001149	0.33
33001174	0.48
33001150	0.52
33022256	0.32
33001472	0.00
33001460	0.27
33001231	0,22
33001319	1.34
33022098	1.52
33001022	0.06

33001344	1.01
33001046	0.06
33001137	1.58
33001228	0.09
